# Protein Biomarkers in Uveitis

**DOI:** 10.3389/fimmu.2020.610428

**Published:** 2020-12-03

**Authors:** Reema Bansal, Amod Gupta

**Affiliations:** Advanced Eye Centre, Post Graduate Institute of Medical Education and Research, Chandigarh, India

**Keywords:** biomarkers, ocular inflammation, proteomics, uveitis, vitreous

## Abstract

The diseases affecting the retina or uvea (iris, ciliary body, or choroid) generate changes in the biochemical or protein composition of ocular fluids/tissues due to disruption of blood-retinal barrier. Ocular infections and inflammations are sight-threatening diseases associated with various infectious and non-infectious etiologies. Several etiological entities cause uveitis, a complex intraocular inflammatory disease. These causes of uveitis differ in different populations due to geographical, racial, and socioeconomic variations. While clinical appearance is sufficiently diagnostic in many diseases, some of the uveitic entities manifest nonspecific or atypical clinical presentation. Identification of biomarkers in such diseases is an important aid in their diagnostic armamentarium. Different diseases and their different severity states release varying concentrations of proteins, which can serve as biomarkers. Proteomics is a high throughput technology and a powerful screening tool for serum biomarkers in various diseases that identifies proteins by mass spectrometry and helps to improve the understanding of pathogenesis of a disease. Proteins determine the biological state of a cell. Once identified as biomarkers, they serve as future diagnostic and pharmaceutical targets. With a potential to redirect the diagnosis of idiopathic uveitis, ocular proteomics provide a new insight into the pathophysiology and therapeutics of various ocular inflammatory diseases. Tears, aqueous and vitreous humor represent potential repositories for proteomic biomarkers discovery in uveitis. With an extensive proteomics work done on animal models of uveitis, various types of human uveitis are being subjected to proteome analysis for biomarker discovery in different ocular fluids (vitreous, aqueous, or tears).

**Graphical Abstract:**
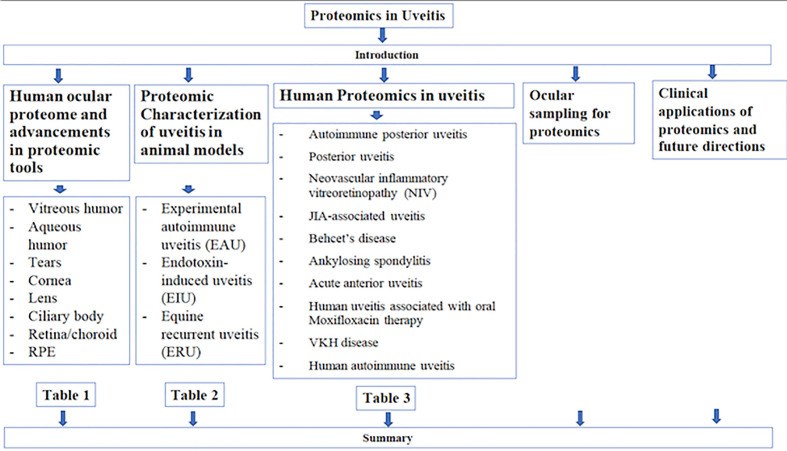
Flowchart of article outline.

## Introduction

Different diseases and their different severity states release varying concentrations of proteins, which can serve as biomarkers. Proteomics is a high throughput technology and a powerful screening tool for serum biomarkers in various diseases that identifies proteins by mass spectrometry and helps to improve the understanding of pathogenesis of a disease. Proteins determine the biological state of a cell. Once identified as biomarkers, they serve as future diagnostic and pharmaceutical targets. While serum, plasma, urine, or sputum samples have been utilized for identifying disease biomarkers, vitreous proteomics is emerging rapidly in ocular diseases (1–3).

Ocular proteomics is a promising field aimed at the advancement of diagnostics and therapeutics of many debilitating ocular diseases (4–6). The enormous protein profile of the intraocular structures, including the intraocular fluids (vitreous and aqueous humor), forms the basis of research into ocular proteomics. Several attempts have been made to characterize the proteome of the vitreous, both in animal models and humans, including healthy and diseased states. Insights into various aspects of an ocular disease have been provided by identification of individual proteins and networks of functionally related proteins with signaling pathways. As a component of the Biology/Disease-driven Human Proteome Project (B/D-HPP), which supports the broad application of state-of-the-art measurements of proteins/proteomes in human diseases, the Human Eye Proteome Project (HEPP) was officially initiated in 2012 to facilitate proteomic studies of the eye (7, 8).

The diseases affecting the retina or uvea (iris, ciliary body, or choroid) generate changes in the biochemical or protein composition of ocular fluids/tissues due to disruption of blood-retinal barrier. Ocular infections and inflammations are sight-threatening diseases associated with various infectious and non-infectious etiologies. Intraocular inflammations (infectious or non-infectious) account for 5%–20% of blindness in the developed world and 25% in the developing world (9, 10). Several etiological entities cause uveitis, a complex intraocular inflammatory disease. These causes of uveitis differ in different populations due to geographical, racial, and socioeconomic variations (10). While clinical appearance is sufficiently diagnostic in many diseases, some of the uveitic entities manifest nonspecific or atypical clinical presentation. Identification of biomarkers in such diseases is an important aid in their diagnostic armamentarium. This review highlights the developments and advances in proteomics of intraocular inflammation or uveitis.

## Human Ocular Proteome and Advancements in Proteomic Tools

### Vitreous Humor

Proteomic analysis of human vitreous humor has revealed a large profile of intracellular and extracellular proteins (11–15). Nourishment of the vitreous humor from the blood vessels of the retina (and ciliary body) and a close proximity to the retina induces several biochemical changes on protein dynamics of the vitreous, which can be detected by a comprehensive proteomic profiling. A number of vitreous fluid proteins have been linked to the etiology of ocular and vitreo-retinal disorders (16, 17). The main components of proteomic studies involve separation of proteins from complex mixtures (fractionation), and their identification by mass spectrometry and data analysis. Proteomic methodologies have evolved from gel electrophoresis (in-gel digestion of proteins) to shotgun proteomics (in-solution digestion of proteins) (18, 19). The diversity present in the ocular cells, tissues, and fluids warrants an adequate sample preparation to produce reliable results from ocular proteomics (20).

Using sodium dodecyl sulphate-polyacrylamide gel electrophoresis (SDS-PAGE), Gao et al. employed liquid chromatography mass spectrometry (LC-MS/MS) and identified 252 proteins in the vitreous of non-diabetic controls (21). Using different prefractionation techniques like one-dimensional (1D) SDS-PAGE or liquid-phase isoelectric focusing (IEF), Aretz et al. identified 1111 proteins (12). The presence of 262 unique proteins in all three patient samples probably represented the constitutive protein pattern of human vitreous. They found striking differences between the vitreous and plasma proteome. Only 27% of their 1111 vitreous proteins were listed as plasma proteins. Contrary to the assumption that most vitreous proteins originate from the plasma, they suggested that the crystalline lens, retina and retinal pigment epithelium (RPE) contribute to the vitreous proteome. The authors also suggested similarities between aqueous and vitreous proteome due to the continuous exchange between the two body fluids.

Presence of high abundant proteins in the vitreous, 80% of which is comprised by albumin and immunoglobulin, limits the detection of low abundant proteins, which is a major challenge with 2D-PAGE experiments. This is partly overcome by employing affinity chromatography for proteomics of body fluids, which improves the detection of low abundant protein detection by depleting the high abundant proteins (22). Kim et al. identified 346 proteins using immunoaffinity depletion and liquid chromatography-Matrix assisted laser desorption and ionization (LC-MALDI-MS/MS) (23). Murthy et al. identified 1,205 proteins in the normal human vitreous humor using LC-MS/MS, of which 682 proteins were reported for the first time (11). Skeie et al. performed a proteomic analysis of four vitreous substructures (anterior hyaloid, vitreous cortex, vitreous core and vitreous base) in three non-diseased human eyes, using multidimensional LC-MS/MS (24). They identified a mean of 2,062 proteins, for each substructure, with 278 proteins being unique to anterior hyaloid, 322 to vitreous cortex, 136 to vitreous core, and 128 to vitreous base. They found many of the vitreous proteins to be originating from the retina, such as rhodopsin and phosphodiesterase, suggesting retina as a potential source of inflammatory mediators. Key pathways and networks were represented, including complement pathway, and damage-associated molecular patterns (DAMPs). Presence of antimicrobial proteins, oxidative stress regulation and energy metabolism proteins distributed throughout the vitreous indicated it to be highly active biologically with dynamic interactions with the surrounding tissues. Further, the various intraocular tissues were protected from infection and oxidative stress by the proteins localized to distinct substructures.

### Aqueous Humor

The human aqueous humor contains about 676 nonredundant proteins, and 328 cytokines, chemokines, and receptors (25). Majority of the proteins have catalytic and enzymatic activity. The proteins are low in concentration (0.1–0.5 mg/ml), and arise from plasma as filtrates, and ciliary body. Richardson et al. used an advanced proteomic approach to characterize the aqueous humor proteome by depleting abundant (albumin) proteins and employing multidimensional protein identification technology (MudPIT) (26). They identified anti-angiogenic, anti-oxidant, and immunoregulatory proteins in the aqueous, with a possible role in supporting avascular tissues.

### Tears

The advancements in MS tools and separation methods have led to an increase in the number of proteins identified in human tears from about 500 to about 1,466 non-redundant proteins, which are secreted by lacrimal glands and epithelial cells of the ocular surface (27). The proteins in high abundance [albumin, lysozyme (LYZ), lactoferrin, and secretory IgA] mask the detection of lower abundant proteins in proteomic studies. Several factors can cause variations in protein profile of tears, such as closed or open eyes, and method of collection (capillary tubes versus Schirmer strips). However, day-to-day variations or fellow eye variations are minimal in tear protein profiles (28).

### Cornea

The involvement of individual layers often in many corneal diseases emphasizes the need for a separate proteomic analysis of different layers of the cornea. Dyrlund et al. identified 3,250 unique Swiss-Prot proteins in human corneas, by using various methods like SDS-PAGE, in-solution digestion, cation exchange chromatography and LC-MS/MS (29). Of these, 2,737 proteins belonged to the epithelium, 1,679 to stroma, and 880 to the endothelium. Of these, 609 (19%) proteins were common to all three layers, with C-reactive protein being one of them. The most abundant proteins included immunoglobulins, albumin, alpha-1-antitrypsin, haptoglobin, complement component 3 and 9, and serotransferrin. Several of the cornea proteins were common to human tears or human aqueous humor, and many are plasma proteins. The study of human corneal proteome has been limited to Fuch’s dystrophy, keratoconus lattice and granular corneal dystrophies. Proteomic investigations of majority of the ocular surface diseases have been performed on tears. In *Fusarium* keratitis, the tear protein profile revealed several differentially expressed proteins (DEPs) in different stages of the infection (early, intermediate and late) (30). Pooled tear samples (five from each group, and from controls) were subjected to DIGE for protein separation, DeCyder software analysis for quantitation, followed by LC-MS/MS. Functional enrichment analysis of DEPs was done by DAVID software.

### Lens

The human lens have the highest protein content in the body. Majority are structural proteins (90%), the crystallins, occurring in three distinct families (α-, β-, and *γ*-crystallin). Due to their long life and minimal protein turnover, the crystallins provide ideal targets to study post-translational modifications (31, 32).

### Ciliary Body

An in-depth proteomic analysis of human ciliary body using LC/MS/MS identified 2815 proteins (33). While many were plasma proteins, 896 proteins were unique to the ciliary body. Pathway analysis showed expression of glycolysis and gluconeogenesis, EIF2 signaling and ubiquitin pathway.

### Retina

About 5042 non-redundant proteins have been identified in the vascular endothelial cells of human retina and/or choroid (34). Of 3,454 proteins that were quantifiable, 498 (14.4%) were differentially expressed among the two cell populations. Angiogenic proteins were identified in both cells, and immunologic proteins were additionally found in retinal cells.

### Retinal Pigment Epithelium

Lipopolysaccharide (LPS) is known to trigger inflammation in the RPE cells. Song et al. used the latest tandem-mass tags (TMTs) label-based approach with high-resolution MS to compare the proteome and phosphoproteome of the LPS-challenged RPE cell line ARPE-19 (human-derived) versus control cells (unexposed) (35). Among the DEPs, the upregulated proteins include those related to amino acid and lipid metabolism, endoplasmic reticulum stress and cell-matrix adhesion. Proteins associated with mitochondrial respiration and cell cycle checkpoint were downregulated. Pathway analysis revealed MAPKK and Wnt/β-catenin signalings. The study demonstrated signals expressed by inflamed RPE, providing insight into various RPE- related disorders.

**Table 1** summarizes the proteomics of human ocular tissues.

**Table 1:** Proteomics of human ocular tissues.

Ocular tissue	Average protein concentration	Proteins in high abundance	Number of proteins identified	Study
**Vitreous humor**	0.5 mg/ml	Albumin and immunoglobulin	1,111	Aretz et al. (12)
			346	Kim et al. (23)
			1,205	Murthy et al. (11)
			2,062	Skie et al. (24)
**Aqueous humor**	0.1–0.5 mg/ml	Albumin	676	Chowdhury et al. (25)
**Tears**		Albumin, lysozyme, lactoferrin and secretory IgA	1,466	Zhou et al. (27)
**Cornea**		Immunoglobulins, albumin, alpha-1-antitrypsin, haptoglobin, complement component 3 and 9, and serotransferrin	3,250	Dyrlund et al. (29)
**Lens**	Highest	Crystallins	–	Kyselova et al. (31)
**Ciliary body**		Plasma proteins	2,815	Goel et al. (33)
**Retina and/or choroid**		Angiogenic proteins	5,042	Smith et al. (34)

## Proteomic Characterization of Uveitis in Animal Models

### Experimental Autoimmune Uveitis or EAU

Animal models of experimental autoimmune uveitis (EAU), a model of non-infectious T-cell mediated autoimmune uveitis, have been used extensively to conduct vitreous proteome studies to explore the immune mechanisms associated with intraocular inflammation. The damage done by oxidative stress induces pathological changes in the retina much before the damage by the infiltrating inflammatory cells, as evident by the mitochondrial retinal DNA damage in early phases of uveitis (36–38). In a mouse model of early EAU, proteomics approach was used to study the mitochondrial protein alterations, using 2D-DIGE and MALDI-TOF MS, followed by validation with Western Blot analysis and real-time PCR (36). Thirteen proteins were differentially expressed in the early EAU mitochondria, as compared to controls, of which nine were upregulated and four were downregulated. The upregulation of crystallins (αA and βB2) and manganese-SOD (MnSOD) indicated their ability to respond to oxidative stress, also offering a protective effect against it. A high metabolic demand and an increased density of mitochondria in the photoreceptors make them susceptible to oxidative stress, which induces an increased expression of αA crystallins. αA crystallin prevents the release of cytochrome c into the cytosol by binding with it, and, hence, prevents damage by inhibiting subsequent photoreceptor apoptosis (39). This study demonstrated for the first time the presence of βB2 crystallins in the photoreceptor mitochondria, where they maintain calcium homeostasis and help stabilize mitochondrial proteins. The early molecular damage in mitochondria was evident by the downregulation of ATP synthase, malate dehydrogenase, calretinin, and guanine nucleotide-binding proteins. The morphology and membrane potential of mitochondria are maintained by ATP synthases. Loss of their activity and reduced cellular ATP levels reflect early pathological changes in EAU. The authors concluded that the alterations in mitochondrial protein expression were induced by mitochondrial oxidative stress in the retina during early phases of uveitis.

Subsequently, the authors demonstrated posttranslational modifications in nine out of the 13 DEPs in the retinal mitochondria during early EAU by using MALDI-TOF (38). These modifications included oxidation (ATP synthase, MnSOD), carbamidomethylation (mtHsp 70, laminin, syntaxin-binding protein, and AT synthase), and pyro-glu modification (αA crystallin). Mitochondrial dysfunction is integral to various pathological diseases, and is caused by altered proteins expression and their posttranslational modification. The latter is associated with events like unfolding, degradation, or aggregation of proteins.

Okuniki et al. used proteomic approach to study the role of retinal autoantigens in spreading the ocular inflammation in human endogenous uveoretinitis (40). After inducing EAU in mice, they detected the presence of autoantibodies against the retinal proteins (antigens) by 2D electrophoresis and western blotting. They identified six new candidate autoantigens in the murine model as the retinal proteins targeted by the autoantibodies using MS, as β-actin (bAct), esterase D (EsteD), tubulin β-2, brain-type creatine kinase (BB-CK), voltage-dependent anion-selective channel protein, and aspartate aminotransferase. They further examined immunoreactivity by ELISA against bAct, EsteD, and BB-CK in human endogenous uveitis [36 Behcet’s disease (BD), 16 Vogt-Koyanagi-Harada (VKH) disease and 17 sarcoidosis patients]. Of these, EstD and BB-CK reacted with sera from BD (25% and 5.6%, respectively), VKH (25% each), and sarcoidosis (17.6% and 38.4%, respectively), suggestive of autoantigenicity in both EAU and human endogenous uveitis. Despite being a melanocyte-specific autoimmune disease, they demonstrated the additional role of autoimmunity to retinal antigens in VKH disease. This study was the first to identify the role of EstD in autoimmunity in human uveitis. The authors believed that the generation of EstD and BB-CK as retinal autoantigens was secondary to the retinal destruction induced by uveitis, which possibly extended the ocular inflammation in endogenous autoimmune uveitis.

To test the hypothesis that different proteins are expressed in different forms of uveitis, Pepple et al. compared the inflamed aqueous humor of two uveitis models of acute inflammation in Lewis rats [EAU-2 weeks, and primed mycobacterial uveitis (PMU)-2 days] with the naïve aqueous humor by proteomics approach using 2D gel electrophoresis and MALDI-TOF (41). A larger amount of ocular fluid is available in rat than mouse. Innate immunity plays a significant role in an animal model of PMU, as the killed mycobacteria are introduced into the vitreous cavity. They found the total protein concentration to be increased in the inflamed aqueous of both uveitis models, as compared to the naïve aqueous, including calprotectin (a heterodimer of S100A8 and S100A9) and apolipoprotein E. Apolipoprotein E had higher levels in EAU than PMU in the aqueous. The vitreous also showed increased levels of S100A8 in both uveitis models, higher in PMU than EAU. Beta-B2-crystallin was markedly decreased in EAU as compared to PMU and naïve eyes, both in the aqueous and vitreous. This finding was in contrast to the previous reports of upregulation of mitochondrial crystallins (both αA and βB2) during early EAU (36). The authors attributed this to the temporal association of βB2 crystallin changes in EAU. The widespread retinal necrosis during “early” EAU caused an increased expression of βB2 crystallin (36), which could not be detected during peak inflammation at “two weeks” owing to retinal cell destruction causing background loss of proteins (41). They found that the overall complexity of the protein constituents was decreased in the inflamed aqueous and increased in the inflamed vitreous (as compared to naïve vitreous) due to breakdown of blood-retinal barrier leading to exudation of serum major proteins into the vitreous.

The patterns of EAU induced by opsin or rhodopsin involve dense cellular infiltration in both retinochoroid and anterior uvea, with rhodopsin being more pathogenic than opsin (42–44). Schalken et al. provided the first evidence of EAU induced by purified rhodopsin in monkeys (44). A dense cellular and humoral response occurred before the onset of EAU, indicating the role of cellular immunity in the pathogenesis of EAU.

### Endotoxin-*Induced Uveitis*

In an acute ocular inflammation model of an animal (endotoxin-induced uveitis or EIU), Bahk et al. used proteomic approach (2D gel electrophoresis and micro LC/LC-MS/MS) to compare the protein infiltration in the vitreous of EIU rats with vitreous of normal rats (45). They found crystallins in intact form in EIU rat vitreous, and truncated βA4- and βB2-crystallin in normal vitreous. They suggested that the crystallins were the predominant proteins involved in EIU, and the specific modifications in crystallin family proteins (such as truncation of C-terminal of β-crystallins) caused progression of EIU-related disease.

### Equine *Recurrent Uveitis*

Equine recurrent uveitis (ERU), a spontaneous model for human autoimmune uveitis, is a blinding, recurrent, lymphocyte-driven, autoimmune disease occurring in horses (46). Proteomic studies of vitreous and retinal samples from ERU eyes versus healthy controls have revealed a list of DEPs, which are associated with maintenance of blood-retinal barrier and immune system (47). In a study by Hauck et al., which compared the retinal proteome of ERU horses with controls (48), glial fibrillary acidic protein (GFAP) was among the several upregulated proteins, which suggested the involvement of retinal Mueller glial cells (RMG) in uveitis. Screening for RMG-specific markers revealed an upregulation of vimentin and GFAP, and downregulation of glutamine synthetase. The subsequent downregulation of pigment epithelium-derived factor (PDEF) by the activated RMGs and expression of pro-inflammatory interferon-gamma indicated a significant role of RMGs in progression of uveitis by triggering interferon-gamma release. Degroote et al. identified 362 spots in the equine lymphocyte proteome by 2D SDS-PAGE (49). When compared with healthy horses, seven DEPs were detected by 2D-DIGE technique, and identified by MS. One (lactotransferrin) was upregulated in ERU lymphocytes while six (voltage-dependent anion-selective channel protein 2, programmed cell death 6-interacting protein, protein tyrosine phosphatase non-receptor type6, glyceraldehyde-3-phosphate dehydrogenase, ezrin and septin 7) were downregulated. A decreased expression of septin 7 in both CD4+ and CD8+ lymphocytes of ERU, with no expression difference in B cells, suggested that the predominant cell types in ERU were the T cells. Septin 7 inhibits cellular proliferation. Its downregulation in ERU activates T cells and promotes inflammation.

Two novel retinal autoantigens were detected in ERU cases by proteomic approach, namely cellular retinaldehyde-binding protein (CRALBP) and malate dehydrogenase (MDH), both with B- and T- cell autoreactivity (50). While MDH induces uveitis only in Lewis rats (and not in horses), CRALBP is uveitogenic in both rats and horses.

**Table 2** summarizes the proteomic characterization of uveitis in animal models.

**Table 2:** Proteomic Characterization of uveitis in animal models.

Type of animal model of uveitis	Tissue studied	Proteins targeted	Inference	Study
Early EAU (mouse)	Retina	Mitochondrial proteins	Demonstrated for the first time the presence of βB2 crystallins in the photoreceptor mitochondria Alterations in mitochondrial protein expression were induced by mitochondrial oxidative stress in the retina during early phases of uveitis.	Saraswathy et al. (36)
EAU (mice)	Retina	Retinal autoantigens	Role of retinal autoantigens in spreading the ocular inflammation in human endogenous autoimmune uveitis	Okuniki et al. (40)
EAU and PMU (2 models of Lewis rats)	Aqueous humor		Overall, the protein constituents were decreased in the inflamed aqueous and increased in the inflamed vitreous (as compared to naïve vitreous) due to breakdown of blood-retinal barrier leading to exudation of serum major proteins into the vitreous.	Pepple et al. (41)
EAU (monkey)		Purified rhodopsin	Role of cellular immunity in the pathogenesis of EAU	Schalken et al. (44)
EIU (rat)	Vitreous		Crystallins were the predominant proteins involved in EIU	Bahk et al. (45)
ERU (horse)	Retina		Upregulation of GFAP suggested the involvement of retinal Mueller glial cells (RMG) in uveitis	Hauck et al. (48)
ERU (horse)	Lymphocytes		The predominant cell types in ERU were the T cells	Degroote et al. (49)
ERU			Two novel retinal autoantigens were detected in ERU (CRALBP and MDH), both with B- and T-cell autoreactivity (uveitogenic)	Deeg et al. (50)

EAU, Experimental autoimmune uveitis; PMU, primed mycobacterial uveitis; EIU, endotoxin-induced uveitis; ERU, Equine recurrent uveitis; CRALBP, cellular retinaldehyde-binding protein; MDH, malate dehydrogenase.

## Human Proteomics in Uveitis

### Vitreous and Autoimmune Posterior Uveitis

Though vitreous proteomics in humans has evolved extensively over the last two decades in healthy eyes as well as in various vitreoretinal disorders, it is still in its infancy in uveitis. Most of the proteomics studies on vitreous samples in uveitis so far have been done in animal models, with only a few studies addressing the proteome of human vitreous in uveitis (51–56).

Several vitreous proteins have been reported to be associated with autoimmune uveitis (51). Kasudhan et al. compared the vitreous fluid proteome of infectious (34 samples) and noninfectious (56 samples) uveitis by using SDS-PAGE (which identified 25 different proteins), 2D electrophoresis (which identified 22 proteins), and matrix-assisted laser desorption and ionization- time of flight mass spectrometry (MALDI-TOF MS) (51). Among the proteins identified by SDS-PAGE, half revealed association with heterotrimeric G-Protein signaling pathway-rod outer segment phototransduction, and the other half were associated with *de novo* purine biosynthesis. Of the proteins identified by 2D electrophoresis, 20% showed association with *de novo* purine biosynthesis. The proteins that were common in both analyses included carbonic anhydrase1 and serpin B3, both of which are associated with acute anterior uveitis (AAU) and autoimmunity (57, 58). About 40% of the proteins were associated with glycolytic pathway. Five of the proteins identified in their study had associations with uveitis: alpha-1-acid glycoprotein (α1-AGP) with acute idiopathic anterior uveitis (59), recoverin with autoimmune uveitis (60), selenium-binding protein 1 with BD (61), perforin with viral uveitis (62, 63), and carbonic anhydrase 1 with AAU (64).

Velez et al. reported the use of proteomics in diagnosing a case of idiopathic uveitis as autoimmune retinopathy by profiling the vitreous fluid biopsy for cytokine expression (52). Seventeen upregulated and 12 downregulated cytokine signals were detected, following which the diagnosis was confirmed by the presence of antibodies against S-arrestin. Administration of targeted therapy led to reversal of visual loss, demonstrating the use of proteomics in personalized medicine. They performed cytokine profiling of vitreous fluid in 15 cases of posterior uveitis and five normal controls. Uveitic vitreous had 60 DEPs, of which insulin like growth factor–binding protein 2 (IGFBP-2), platelet-derived growth factor receptor β polypeptide (PDGFRb), interleukin 23 (IL-23), IL-17R, IL-1 receptor I (IL-1RI), nerve growth factor (b-NGF), bone morphogenic protein 4 (BMP-4), tissue inhibitors of metalloproteinase 1 and 2 (TIMP-1 and TIMP-2), and stem cell factor (SCF) were expressed in vitreous of all uveitis cases.

Characterization of uveitis proteome has translational significance and relevance for personalized proteomics (53). Beyond their diagnostic indications, the use of proteomics of ocular fluids is extending into the clinics for personalized management of patients. Detection of a cytokine signature common to all 15 cases with different forms of uveitis [idiopathic posterior uveitis, viral endophthalmitis, autoimmune retinopathy, HLA-B27 uveitis, multifocal choroiditis, and neovascular inflammatory vitreoretinopathy (NIV)] opened the possibility of targeting different diseases with the same therapy (52). In eyes with NIV, an autoinflammatory, hereditary disease with blinding outcome despite intensive therapy, real time proteomics of liquid biopsies in various stages of the disease enabled detection of dynamic events during intraocular inflammation (54). Vitreous profiling was done to analyze 200 cytokine-signaling proteins by an antibody array by comparing NIV (8 eyes) and non-NIV (4 eyes) control eyes. By cytokine array, 64 DEPs were detected (61 upregulated and 3 downregulated). The TNF-α levels were found normal in all NIV eyes. This explained the failure of these eyes to respond to previous treatment with conventional immunosuppression with an anti–TNF-α agent (infliximab). Detection of elevated levels of vascular endothelial growth factor (VEGF) by proteomic analysis guided the anti-VEGF therapy in NIV eyes with successful outcomes, without the need for vitreous surgery for hemorrhagic complications. Pathway analysis revealed significant representation of the mTOR and class I PI3K signaling pathways. Considering the mTOR effectors present in NIV vitreous, the authors redirected the previous ineffective oral methotrexate therapy to intravitreal methotrexate therapy to target NIV-specific T cells. A dramatic reduction in anterior chamber inflammation led to the use of intravitreal methotrexate in routine clinics as an adjunct for perioperative management of NIV patients, also avoiding the corticosteroid-related side effects. Recurrent fibrosis could be prevented by anti-IL-6 therapy.

Velez et al. utilized vitreous proteomic strategy to personalized medicine, emphasizing protein biomarkers for a therapeutic potential, rather than as diagnostic biomarkers (52). They recommend the vitreous protein profiling for a therapeutic approach, to identify the best therapy by targeting proteins that are present in the vitreous, and avoiding the ones that are not found in the vitreous.

### Intermediate Uveitis

Sepah et al. recently reported proteomics screening of vitreous samples of three patients (four eyes) with intermediate uveitis (IU) to discover biomarkers and therapeutic targets, suggesting the role of both innate and adaptive immunity in IU (56). The LC-MS/MS approach identified 233 DEPs (103 proteins upregulated 130 downregulated) among IU and control samples. Fibronectin (FN) was among the proteins with highest expression and glutathione synthetase (GSS) among the least expression in IU samples. Known for organizing collagen and extracellular matrix components, an upregulated FN in IU may be responsible for snowballs formation, a finding typical of IU. The downregulated GSS in IU indicates a reduced regulation of oxidative stress. Ingenuity Pathway Analysis (IPA) revealed complement system, coagulation system, glycolysis and IL-12 (with 22 inflammatory mediators) signaling in macrophages among the most represented pathways. The most significantly activated regulators of these pathways were myeloid differentiation primary response protein (MYD88) and IL-23. As both promote myeloid cell recruitment and differentiation, myeloid cell recruitment was found to be an upstream pathway in IU pathogenesis. The upregulated IL-23 in IU vitreous helped CD4+ T cells to differentiate into IL-17 helper T cells, which promote the release of pro-inflammatory mediators. The IL-23/IL-17 pathway is integral to many systemic autoimmune diseases, and its expression in IU suggests the autoimmune pathologic drive in IU. Also, it offers the potential to be a therapeutic target in IU by the biologics.

### Juvenile Idiopathic Arthritis–Associated Uveitis

Several biomarkers (S100, S100A8, S100A9, and S100A12 proteins) have been associated with various aspects of systemic manifestations of Juvenile Idiopathic Arthritis (JIA), in predicting the phenotype, severity, and progression of the disease, for predicting flare of the disease, higher risk of poor response to methotrexate treatment (two single nucleotide polymorphisms or SNPs in the ATIC gene and one SNP in ITPA gene), predicting response to treatment (S100), and disease relapse (high S100A12 and MRP8/14) with six months of discontinuing treatment (65). Similar challenges occur in uveitis associated with JIA, prompting the need for biomarker discovery from ocular fluids.

In the first study of proteomics of aqueous humor of children with uveitis, Ayuso et al. investigated the aqueous and serum samples (paired) of patients with JIA-associated uveitis (62 patients), using SELDI-TOF MS, and compared with silent chronic anterior uveitis (non-infectious) (7 patients), other uveitis entities (27 patients), and non-inflammatory controls (15 patients; pediatric cataract or glaucoma) (66). They found significant differences in the protein profile of JIA-associated uveitis, when compared to other uveitis entities and non-inflammatory controls, but similar protein compositions in aqueous humor of patients with silent chronic AU and JIA-associated uveitis. They hypothesized that patients with chronic silent AU were a subset of JIA, primarily manifesting with uveitis, but without rheumatological signs. A significantly higher concentration of transthyretin (TTT) in aqueous humor of patients with silent chronic AU and JIA-associated uveitis as compared to other groups led to its identification as a potential intraocular biomarker of JIA-associated uveitis. In human eye, TTR is produced by the RPE, and plays a role in the transport of thyroid hormones and retinol-binding protein (RBP). Further, TTT was reported as one of the proteins upregulated in synovial fluid of rheumatoid arthritis patients (67). Subsequently, Clement et al. used biochemical and proteomic approaches to demonstrate an autoimmune response to TTT in JIA, suggesting its role in the pathogenesis of JIA as an autoantigen (68).

This was followed by a pilot study of tears to evaluate for biomarkers in children with chronic non-infectious AU, using LC-MS/MS (69). Tears were collected from seven patients using Schirmer strips: three with JIA-associated uveitis (JIA-U), and four with idiopathic chronic anterior uveitis (I-CAU). Various cytokines (S100A8, S100A9, TTR, sCD14, LAP3, and SAA1) that are present in aqueous humor of JIA-U, were detected in tears of all JIA-U and I-CAU children, with a higher expression of S100A8, sCD14, and SAA1 in JIA-U tears, and a lower expression of S100A9, TTR, LAP3, and MIF in JIA-U tears, as compared to I-CAU tears. The authors demonstrated a similar cytokine profile of tears and aqueous in children with CAU. The differential expression of several proteins in tears of JIA-U and I-CAU reflected intrinsic differences between the two phenotypically similar entities.

To study the autoimmune target structures in JIA-associated uveitis, Busch et al. employed 2D-PAGE, western blotting and MS to analyze the binding patterns of serum autoantibodies to proteomes isolated from porcine ocular tissues (iris, ciliary body, and retina) (70). A broad range of serum antibodies were found in JIA-U, mostly directed against iris antigens (vimentin, tubulin beta chain, ATP synthase subunit beta, actin, and L-lactate dehydrogenase B chain. With iris being the major site of inflammation in JIA-U, these findings support the role of autoantibodies in immunopathology of JIA-associated uveitis.

### Behcet’s Disease

BD is a chronic, autoinflammatory disease with multisystem involvement. Uveitis is chronic, recurrent, bilateral, and sight-threatening. The lack of clinical or diagnostic biomarkers makes it imperative to explore prognostic biomarkers to predict the disease progression and response to therapy. Hu et al. identified CTDP1 (RNA polymerase II subunit A C-terminal domain phosphatase) as a novel BD autoantigen using HuProt arrays (71).

In BD patients with active uveitis, serum samples were subjected to proteomic analysis using magnetic beads, MALDI-TOF-MS, and bioinformatics tool, and compared with non-BD patients (VKH disease and healthy controls) (72). Thirty-nine DEPs were detected, which indicated the involvement of unidentified proteins, and a broad change in complemented immunoglobulins and autoantibodies in progression of BD. This differential serum proteomic profile of BD (with 39 differential m/z peaks) was used to construct a classification algorithm, based on which BD could be successfully distinguished from VKH and controls using a panel of six m/z peaks. This diagnostic model had a sensitivity of 84.62% and specificity of 90.48% in diagnosing BD, and a sensitivity of 90% and specificity of 100% in diagnosing VKH disease. The discovered biomarkers also aided in determining the stages of BD.

The most recent breakthrough in proteomics of uveitis was reported in tears of BD patients with unilateral relapse of uveitis (73). Fifteen patients with unilateral relapse of BD-associated uveitis (BDU) were enrolled. Tears were collected from both eyes (the diseased eye with relapse of uveitis and fellow quiescent eye), and subjected to data-independent acquisition (DIA) proteomics for protein profiling. Of 1,797 proteins detected, 51 were differentially expressed in uveitic and quiescent eyes, some of which were related to inflammation or immunity. In uveitis-relapsed eyes, α1-AGP, an immunomodulatory protein, was increased. Annexin A1 (ANXA1), an anti-inflammatory protein, and LYZ were decreased. The role of ANXA1 in innate and adaptive immunity is multifunctional and involves resolution of inflammation. This study strengthened the role of tear proteomics as potential sources of biomarkers for uveitis and established α1-AGP and ANXA1 as potential biomarkers for monitoring of uveitis in BD.

## Ankylosing Spondylitis

Sera from ankylosing spondylitis (AS) or other autoimmune disease patients were subjected to human protein microarray analysis (74). A significantly higher level of anti-prefoldin subunit 5 (PFDN5) antibodies were found in sera of AS patients with uveitis, as compared to AS without uveitis. To further assess the pathogenic role of this biomarker identified in AS with uveitis, the authors conducted an animal study involving curdlan-induced SKG mice model, which developed uveitis and peripheral arthritis, compared to PBS-treated SKG mice. Half of them developed anterior uveitis at week 8, while all developed uveitis (histologically confirmed) at week 16. The ocular lesions demonstrated apoptosis and an increased PFDN5 expression, and the sera showed increasing levels of anti-PFDN5 antibodies over time. PFDN5 also revealed a protective role against apoptosis of retinal cells, preventing cell death in uveitis.

### Acute Anterior Uveitis

Tear samples from five patients with unilateral AAU were subjected to proteomics using LC-MS/MS to detect any ipsilateral changes in tear proteome, with fellow unaffected eyes serving as controls (75). Of 242 identified proteins in both groups of eyes, 32 tear proteins were detected in all five patients. Nine proteins were upregulated and eight downregulated in uveitis eyes as compared to normal eye. The diseased eyes showed a significant increase in Apolipoprotein B mRNA editing enzyme catalytic subunit 3A (APOBEC3A), and a significant decrease in transglutaminase 2 (TGM2). The top canonical pathways identified by IPA to be affected in tears included the liver X receptor/retinoid X receptor (LXR/RXR) and the farsenoid X receptor/retinoid X receptor (FXR/RXR). IL-6 and tumor necrosis factor (TNF) were identified as possible upstream regulators. The study showed that the tears expressed ipsilateral proteomic changes in unilateral AAU, which were detectable by MS.

### Human Uveitis Associated With Oral Moxifloxacin Therapy

Oral moxifloxacin therapy is associated with bilateral panuveitis with anterior chamber pigment dispersion (76, 77). In a case report by Hinkle et al., aqueous humor samples were obtained from both eyes of a patient with bilateral uveitis-like syndrome with pigment dispersion during acute phase, and one eye of a normal subject who consented for aqueous harvest prior to cataract surgery. Moxifloxacin concentration was tested by multiple reaction monitoring (MRM) MS. Proteomic analysis was done using shotgun approach *via* LC-MS/MS (78). Eighteen days after completion of oral intake, aqueous humor demonstrated higher than expected levels of moxifloxacin. The aqueous of affected eyes revealed 33 proteins, and that of unaffected (control) eye showed 32 proteins. Ten proteins were significantly downregulated in uveitis eyes.

### VKH Disease

The role of CD4+ T cells in autoimmune diseases including VKH disease is well known. Mao et al. used label-free quantitative proteomic strategy to detect DEPs between VKH patients and healthy controls (79). They tested blood samples of five active VKH patients and five healthy controls. Three hundred fifty-four peptides were differentially expressed between the two groups, of which 102 could be sequenced, corresponding to 64 proteins. A difference of at least 1.5-fold was seen in 30 proteins between VKH patients and normal controls. Among these, the expression of two proteins, CD18 and AKNA, was more than three-fold lower in active VKH patients compared to controls. These proteins were further validated in other five active VKH patients and six normal subjects by Western blot assay. As CD18 induces apoptosis of human T cells, its reduction in active VKH disease suggests a reduced inhibition of CD4+ T cell apoptosis. The observations of this study open the research on the role of these proteins in influencing apoptosis of CD4+ T cells and cytokine expression.

### Human Autoimmune Uveitis and CRALBP

The role of CRALBP as an autoantigen is well known in ERU. Its role in human autoimmune uveitis was tested by Deeg et al., using 2D western blotting, followed by SDS-PAGE and MS for identification of immunoreactive proteins (80). Sera from 56 uveitis patients and 23 healthy subjects (controls) were used. The normal equine retinal proteome was used as a source of antigen in 2D Western blots, where human uveitis sera were made to react with CRALBP. CRALBP expression pattern in normal eyes was evaluated by using six human donor eyes (normal with no signs of uveitis), displaying a strong CRALBP expression in Mueller glial cells. Anti-CRALBP autoantibodies were found in 30 (54%) out of 56 uveitis sera, and in four (17.4%) out of 23 control sera (p < 0.01), suggesting the role of CRALBP as a potential autoantigen in human autoimmune uveitis.

**Table 3** summarizes the proteomics of various types of human uveitis.

**Table 3:** Proteomic studies in various types of human uveitis.

Type of uveitis	Tissue studied	Differentially expressed proteins	Enriched pathway	Study
Autoimmune posterior uveitis	Vitreous	alpha-1-acid glycoprotein, recoverin, selenium-binding protein 1, perforin, carbonic anhydrase 1	glycolytic pathway	Kasudhan et al. (51)
Posterior uveitis	Vitreous	insulin like growth factor–binding protein 2 (IGFBP-2), platelet-derived growth factor receptor β polypeptide (PDGFRb), interleukin 23 (IL-23), IL-17R, IL-1 receptor I (IL-1RI), nerve growth factor (b-NGF), bone morphogenic protein 4 (BMP-4), tissue inhibitors of metalloproteinase 1 and 2 (TIMP-1 and TIMP-2), and stem cell factor (SCF)		Velez et al. (52)
Neovascular inflammatory vitreoretinopathy (NIV)	Vitreous	Elevated vascular endothelial growth factor (VEGF) levels; normal TNF-α levels	mTOR and class I PI3K signaling pathways	Velez et al. (54)
Intermediate uveitis	Vitreous	233 DEPs (103 upregulated and 130 downregulated); Fibronectin, glutathione synthetase	complement system, coagulation system, glycolysis and IL-12 (with 22 inflammatory mediators) signaling in macrophages	Sepah et al. (56)
Juvenile Idiopathic Arthritis (JIA)-associated uveitis	Aqueous humor and serum (paired samples)	Transthyretin (TTT)	Biomarker in JIA	Ayuso et al. (66)
Juvenile Idiopathic Arthritis (JIA)-associated uveitis	Tears	Cytokines (S100A8, S100A9, TTR, sCD14, LAP3 and SAA1)		Angeles-Han et al. (69)
Behcet’s disease	Serum	39 DEPs; CTDP1 (RNA polymerase II subunit A C-terminal domain phosphatase) as a novel BD autoantigen in Behcet’s disease		Hu et al. (71)
Behcet’s disease	Tears	Alpha-1-acid glycoprotein 1 (α1-AGP), Annexin A1 (ANXA1)	Innate and adaptive immunity	Liang et al. (73)
Ankylosing spondylitis	Serum	anti-prefoldin subunit 5 (PFDN5) antibodies		Kwon et al. (74)
Acute Anterior Uveitis	Tears	17 DEPs; Apolipoprotein B mRNA editing enzyme catalytic subunit 3A (APOBEC3A) (upregulated), transglutaminase 2 (TGM2) (downregulated)	liver X receptor/retinoid X receptor (LXR/RXR) and the farsenoid X receptor/retinoid X receptor (FXR/RXR)	Eidet et al. (75)
Human uveitis associated with oral Moxifloxacin therapy	Aqueous humor	higher than expected levels of moxifloxacin		Hinkle et al. (78)
Vogt-Koyanagi-Harada disease	Blood	30 DEPs; CD18 and AKNA	Apoptosis of human T cells	Mao et al. (79)
Human autoimmune uveitis	Serum	Anti-CRALBP autoantibodies	role of CRALBP as a potential autoantigen in human autoimmune uveitis	Deeg et al. (80)

DEPs, Differentially expressed proteins.

## Ocular Sampling Techniques for Proteomics

Procurement of surgical specimens is critical for proteomic analysis, along with their handling and storage. The invasive nature of vitreous and aqueous sampling restricted the research in human subjects till few decades ago. Skeie et al. reported comparable yields of vitreous needle biopsies and vitrectomy (cutter) biopsies (81). They compared 23-gauge needle method with 23-gauge vitreous cutter method performed sequentially for vitreous biopsy in 10 eyes. The UV absorbance spectroscopy revealed equivalent protein concentrations in paired samples. The SDS-PAGE revealed similar molecular weight protein profiles, and LC-MS/MS identified equivalent peptides in paired samples, with only a few proteins and peptides differing in concentration. The aspiration method has the advantages of being safe and reproducible, yields undiluted vitreous and can be performed in an office-based setting under local anesthesia (82, 83). However, the limited sample volume is a major limitation for performing large-scale proteomic studies. On the other hand, vitreous biopsy cutter method yields adequate volume of sample and reduces the chances of hypotony. It is, however, more invasive and requires vitreous surgery in the form of pars plana vitrectomy (PPV) in an operating room. Vitreous needle biopsy by a fine needle aspiration was the least complex and most followed method, until the advent of microincision vitreous surgery (MIVS). The liquid vitreous specimens transported to the laboratory are centrifuged to separate the cellular debris (pellet) and supernatant. While pellet is subjected to DNA-based molecular tests, the supernatant is cryopreserved at −80°C for future research studies, including proteomics. Though the safety and efficacy of PPV is now well established in uveitis in the era of MIVS (84, 85), their contribution toward proteomic quality of vitreous samples is yet to be addressed.

Sampling of aqueous humor in uveitis is indicated in anterior uveitis, or in cases where PPV is not indicated. The human aqueous humor of healthy controls is typically sampled during cataract surgery. The study eye with uveitis usually undergoes an aqueous biopsy or an anterior chamber tap in an operating room (25, 86). A 25- or 26- or 30- gauge needle is inserted in to the anterior chamber at the limbus through the peripheral cornea through a paracentesis tract, and about 100 µl of the aqueous fluid can be aspirated until the shallowing of anterior chamber starts to occur.

Tear protein profiles have been characterized in several inflammatory and non-inflammatory ocular disorders, using MALDI-TOF, surface-enhanced laser desorption/ionization time-of-flight mass spectrometry (SELDI-TOF-MS) and LC-MS. A relatively high concentration of proteins in the tears along with the non-invasive nature of tear sample collection make tears a potential source for biomarker discovery in proteomic studies (87–89).

## Clinical Applications and Future Directions in Proteomics of Uveitis

The discovery of biomarkers in uveitis is showing a promising role in clinical practice. The diagnosis of autoimmune retinopathy, a rare uveitis entity with a diagnostic and therapeutic challenge, was made possible in a case of idiopathic uveitis by cytokine profiling of the vitreous, which demonstrated the presence of antibodies against S-arrestin (52). Normal levels of TNF-α in the vitreous of NIV eyes explained the reason for failure of NIV eyes to respond to treatment with anti–TNF-α agent (54). In the absence of clinical or diagnostic biomarkers, the identification of protein biomarkers in serum and tears of BD patients provided valuable clues in differentiating this disease from another autoimmune disease (VKH disease), helped in staging the disease based on serum biomarkers, and guided in monitoring therapy for uveitis based on tear biomarkers (72, 73). Different forms of autoimmune uveitis can be targeted with the same therapy with the identification of a protein/cytokine signature common to all of them (52).

A differential protein expression in the intraocular fluids of patients with two phenotypically similar entities has significant clinical implications. Transthyretin (TTT), a potential intraocular biomarker, found in the aqueous humor of silent chronic AU and JIA-associated uveitis patients, provided a common underlying etiology between the two conditions (66).

Tubercular uveitis, a common uveitis entity in endemic countries, poses several clinical and diagnostic challenges, leading to a delay in initiating targeted therapy. The use of proteomics to provide biomarkers in differentiating this disease from other forms of infective and non-infective uveitis is strongly indicated and is a subject of concern.

With the major goal of identifying biomarkers of a disease, proteomics provides information about the changes in protein expression levels between the diseased and normal states, posttranslational modifications, and response to treatment. A new insight into the pathogenesis and course of the disease is obtained by identification of DEPs, and their pathway analysis. The protein constituents identified in a diseased eye can serve as potential targets for therapeutic intervention by the drugs that are available and accessible. Also, proteomics provides information about the drugs to be avoided. Besides their impact on diagnosis and treatment of a disease, proteomics may also have implications in gene therapy trials.

## Summary

Considering the various EAU and EIU studies, crystallins may serve as suitable targets for adjunctive therapy in acute phases of uveitis, as also seen by their role in apoptosis and neuroprotection. The ocular proteome in acute uveitis is likely to differ considerably from that of chronic uveitis, addressing the need for exploring and targeting different proteins in different stages of uveitis. With a potential to redirect the diagnosis of idiopathic uveitis, ocular proteomics provide a new insight into the pathophysiology and therapeutics of various ocular inflammatory diseases. Tears, aqueous, and vitreous humor represent potential repositories for proteomic biomarkers discovery in uveitis. Determining the molecular pathways enriched in uveitis helps in considering the drugs that are available and could readily target the pathways, rather than finding new drugs.

## Author Contributions

RB has contributed in literature search, and manuscript writing. AG has contributed in manuscript writing and refining. All authors contributed to the article and approved the submitted version.

## Conflict of Interest

The authors declare that the research was conducted in the absence of any commercial or financial relationships that could be construed as a potential conflict of interest.
